# Slow Freezing Coupled Static Magnetic Field Exposure Enhances Cryopreservative Efficiency—A Study on Human Erythrocytes

**DOI:** 10.1371/journal.pone.0058988

**Published:** 2013-03-08

**Authors:** Chun-Yen Lin, Po-Li Wei, Wei-Jen Chang, Yung-Kai Huang, Sheng-Wei Feng, Che-Tong Lin, Sheng-Yang Lee, Haw-Ming Huang

**Affiliations:** 1 School of Dentistry, College of Oral Medicine, Taipei Medical University, Taipei, Taiwan; 2 Graduate Institute of Biomedical Materials and Tissue Engineering, College of Oral Medicine, Taipei Medical University, Taipei, Taiwan; 3 Department of Surgery, School of Medicine, College of Medicine, Taipei Medical University, Taipei, Taiwan; 4 Division of General Surgery, Department of Surgery, Taipei Medical University Hospital, Taipei Medical University, Taipei, Taiwan; 5 School of Oral Hygiene, College of Oral Medicine, Taipei Medical University, Taipei, Taiwan; Iwate University, Japan

## Abstract

The aim of this study was to assess the cryoprotective effect of static magnetic fields (SMFs) on human erythrocytes during the slow cooling procedure. Human erythrocytes suspended in 20% glycerol were slowly frozen with a 0.4-T or 0.8-T SMF and then moved to a −80°C freezer for 24 hr. The changes in survival rate, morphology, and metabolites of the thawed erythrocytes were examined. To understand possible cryoprotective mechanisms of SMF, membrane fluidity and dehydration stability of SMF-exposed erythrocytes were tested. For each test, sham-exposed erythrocytes were used as controls. Our results showed that freezing coupled with 0.4-T or 0.8-T SMFs significantly increased the relative survival ratios of the frozen-thawed erythrocytes by 10% and 20% (*p*<0.001), respectively. The SMFs had no effect on erythrocyte morphology and metabolite levels. However, membrane fluidity of the samples exposed to 0.8-T SMF decreased significantly (*p*<0.05) in the hydrophobic regions. For the dehydration stability experiments, the samples exposed to 0.8-T SMF exhibited significantly lower (*p*<0.05) hemolysis. These results demonstrate that a 0.8-T SMF decreases membrane fluidity and enhances erythrocyte membrane stability to resist dehydration damage caused by slow cooling procedures.

## Introduction

Although frozen erythrocytes have been used in transfusion medicine for many years, researchers continue to focus on improving cryopreservation methods. It is well known that optimal cooling rates for cell cryopreservation vary between different cell types [1]. This is because optimal cooling protocol for cryopreservation is strongly dependent on the composition and function of the cell membrane being frozen [2]. Several investigators used water transport models to theoretically predict the optimal cell cooling method [2], [3]. Their results showed that cells with lower membrane permeability exhibited greater survival when frozen at slow cooling rates [4], [5]. Since membrane permeability of erythrocytes is much higher than that of other cells, the optimal cooling rate for erythrocytes is reported to be greater than 1000°C/min, which is almost 1000-fold higher than that for relatively impermeable stem cells (1°C/min) [1], [2]. This unique characteristic makes erythrocyte freezing protocol more complicated than that for stem cells. To overcome this problem, freezing erythrocytes in a high concentration of glycerol while using a slow cooling procedure was proposed for clinical erythrocyte cryopreservation [6].

When cells are frozen at a cooling rate lower than their specific optimal cooling condition, more ice crystals form in extracellular areas. This effect raises the concentration of extracellular solutes in the unfrozen fraction and causes cell dehydration and volume reduction due to exosmosmosis [7]–[9]. This cell dehydration phenomenon also induces large mechanical stresses on the cell membrane during freezing [10], and causes physical disruption of the cells or loss cell membrane integrity after thawing [11]. Accordingly, this membrane destabilization brought about by excessive dehydration is considered the primary cryo-injury of frozen erythrocytes during the slow cooling procedure [12]–[14].

To protect cells under a slow cooling rate, permeable cryoprotectants such as glycerol and dimethyl sulfoxide (DMSO) are used to reduce ice formation, decrease the concentration of damaging solutes, increase the unfrozen fraction [1], [2], sustain a certain cells volume [9], [15], and stabilize cell membranes [16]. Although 10% DMSO provides good cryoprotective efficiency for mononuclear cells in bone marrow and umbilical cord blood, it is harmful for freezing erythrocytes. The reason for this phenomenon is that erythrocyte membrane permeability is about two orders of magnitude higher than other cell types. This unique property causes erythrocytes to dehydrate more rapidly during the freezing process [17].

As mentioned above, a high concentration (40% w/v) of the low toxicity cryoprotectant glycerol was used for clinical erythrocyte cryopreservation [6]. However, the addition of such a high concentration of glycerol results in 17-fold higher osmolality (5,000 mOsm/kg) than the normal physiological range [18]. Therefore, stepwise loading and removal of the high concentration of glycerol is needed to prevent exceeding the osmotic tolerance of erythrocytes [9]. Although this freezing method has been approved and has been routinely used in blood banks for many years, the processes of glycerolization and deglycerolization are still time consuming and increase the cost of each erythrocyte unit. Accordingly, new methods of erythrocyte cryopreservation are receiving considerable attention. Although several scholars have demonstrated that high concentrations of glycerol could be replaced by oligosaccharide [19], trehalose [20], or biopolymers [21], the problem of reducing the glycerol concentration is still a limitation in clinical erythrocyte cryopreservation.

The major composite component of cell membranes, phospholipid, is a molecule that exhibits a highly diamagnetic anisotropic susceptibility. Phospholipids can be orientated by the torque force of a static magnetic field (SMF) [22]–[24]. SMFs affect the alignment of phospholipids in the cell membrane such that the membrane rigidity is increased, while membrane fluidity is decreased [25]–[28]. Interestingly, Ali [29] reported that erythrocyte membrane permeability is significantly decreased when erythrocytes are exposed to the SMF of an MRI scanner, and that the SMF effects on erythrocytes are correlated to the diamagnetic anisotropic susceptibility of erythrocyte membrane.

Although the efficiency of erythrocyte cryopreservation is affected by the physical properties of the cell membrane which are influenced by an SMF, whether or not SMF exposure can be used to improve the efficiency of erythrocyte cryopreservation is still unknown. For this study, the hypothesis is that SMF exposure during the freezing process will enhance cell membrane stability, thus improving the survival rate of thawed erythrocytes frozen in a low concentration of glycerol using a slow cooling procedure.

## Materials and Methods

All experimental protocols presented in this study were approved by the Committee on Human Research, Taipei Medical University.

### Sample preparation and freezing

Venous blood was collected from 16 healthy adult volunteers, 9 males and 7 females, with ages ranging from 25 to 35 years. To prepare the packed erythrocytes, the whole blood was mixed with a 7/50 volume of citrate phosphate dextrose adenine (CPDA)-1 anticoagulant solution and centrifuged at 330× *g* for 14 min to remove the platelet-rich plasma and leukocytes. The packed erythrocytes were stored at 4°C for no longer than 6 days. Before the experiments, the packed erythrocytes were washed three times with phosphate buffered saline (PBS) by repeated resuspension and centrifugation at 520× *g* for 5 min. Then the samples were adjusted to a hematocrit (Hct) of 75% (v/v) in PBS as the erythrocyte suspension. Before the slow cooling procedure, the glycerolized samples were prepared by slowly adding four parts of 35% (w/v) glycerol into three parts of the erythrocyte suspension to achieve a final glycerol concentration of 20% (w/v). Each 0.5 ml of the glycerolized sample was loaded into a 0.6-ml tube.

**Figure 1:**
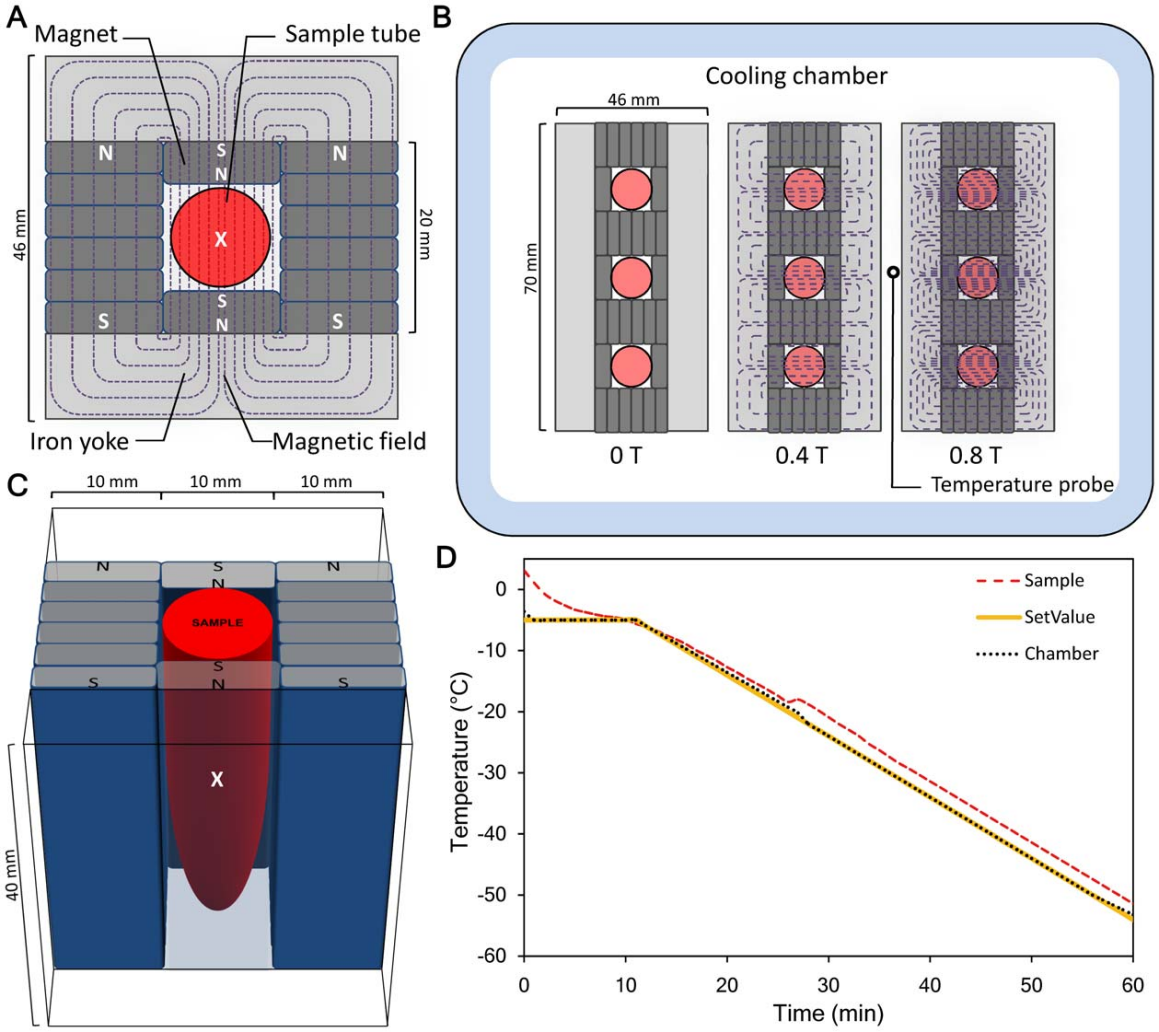
Top view of the experimental setup used for SMF-freezing process. (**A**) The erythrocyte sample tube was surrounded with NdFeB magnets and iron yokes. The chained lines illustrate the magnetic field lines of the sample chamber. (The white cross mark demonstrates the position of the thermometer which used for the sample temperature detection.) (**B**) Three sample tubes were arranged in a row as a sample group. Sample tubes of the three SMF groups with flux densities of 0 T, 0.4 T, and 0.8 T were placed in a cooling chamber of a computer controlled freezer. (Black circle mark: the position of the platinum resistance thermometer which used for detecting chamber temperature.) (**C**) A three-dimensional illustration of the sample chamber. The Gauss meter was placed at the center position of the tube (cross mark) to measure the magnetic flux density. (**D**) The solid curve shows the temperature setting of the computer program. The measured temperatures in the cooling chamber and sample tube were plotted as dotted and dashed lines, respectively.

As shown in Figures 1A and 1B, the sample chambers used in this study were made using NdFeB permanent magnets with a pair of iron yokes. Non-magnetized NdFeB blocks were purchased from a company (N52; Ney Hwu Magnetism Material, Taipei, Taiwan). They were then magnetized in the lab to different strengths using an impulse magnetizer (Ney Hwu Electrical, Taipei, Taiwan). The iron yolk was built by stacking three steel plates to a total thickness of 12 mm. The plates were made of low-carbon soft steel and display good SMF permeability (composition: C 0.04%, Mn 0.26%, P 0.019%, S 0.004%, Si 0.01%, and Al 0.052%)(China Steel, Kaohsiung, Taiwan). When a magnetic field of 5000 A/m was applied to this steel, magnetic flux density was larger than 1.7 Tesla (T). The sample chambers with different SMF strengths were established by using magnets with various magnetic flux densities. For the experimental groups, the magnetic flux densities were set to 0.4 and 0.8 T. For the control group, a 0 T environment was prepared by substituting non-magnetized NdFeB blocks for the magnets. All SMF flux densities were measured with a Gauss meter (Model 5070; FW Bell, Orlando, Florida, United States) at the center position of sample chambers, as shown in Figure 1C.

During the slow cooling procedure, glycerolized samples were placed in the sample chambers and cooled in a computer controlled freezer designed in our lab (Figure 1B). This freezer was cooling by liquid nitrogen with circulating air flow. Temperatures at the chamber and sample tube were monitored using a platinum resistance temperature detector. A two-step program was used to freeze the samples. The initial temperature was set at −5°C and maintained for 10 min. The temperature was then continuously cooled to −55°C at a rate of 1°C/min (Figure 1D). Immediately after the freezing program, the frozen samples were transferred to a −80°C mechanical freezer without an SMF. Since the aim of this study focused on the effect of SMF during the erythrocyte freezing process, the frozen samples were stored at −80°C for 24 hr then the frozen samples were quickly thawed in a 37°C water bath for 1 min.

### Erythrocyte survival test

Erythrocyte survival rates after freezing and thawing were assessed using a hemolysis test. In this test, total and supernatant hemoglobin (Hb) were determined using Drabkin's reagent (Sigma-Aldrich, St Louis, Missouri, United States) with an absorbance measurement at 540 nm (Model 2020; Anthos Labtec Instruments, Wals, Salzburg, Austria). The Hct of the thawed sample was measured using the spun capillary method. The percentage of hemolysis [30] and survival rate were calculated using the following formulas:
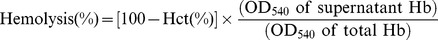


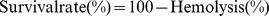



To normalize the data, the relative survival ratio was defined as the ratio between the survival rate of the SMF-exposed erythrocyte samples and the sham-exposed controls from the same volunteer.

### Morphology examinations

The thawed erythrocytes of the experimental and control groups were observed using an optical microscope (TS 100; Nikon, Chiyoda, Tokyo, Japan) coupled with a digital camera (SPOT Idea; Diagnostic Instruments Inc, Sterling Heights, Michigan, United States). In addition, the corpuscular volume distribution and the mean of corpuscular volume (MCV) of thawed erythrocytes were analyzed using an automatic cell analyzer (Scil Animal Care Company, Viernheim, Hessen, Germany).

### Erythrocyte metabolite assay

To understand the functions of the thawed erythrocytes after the freezing process, the concentrations of adenosine-5'-triphosphate (ATP) and 2,3-diphosphoglycerate (DPG) were measured [31]. To prepare the samples for these tests, each thawed erythrocyte sample was added to 1 ml of perchloric acid (0.6 M) on ice for 10 min, then centrifuged at 12,000× *g* for 5 min, while the supernatant was neutralized with 2.5 M potassium carbonate on ice for 60 min. After centrifugation, the concentrations of ATP (BioVision, Milpitas, California, United States) and 2,3-DPG (Roche Diagnostics, Indianapolis, Indiana, United States) in the final extract were measured.

### Membrane fluidity test

To perform the membrane fluidity test, erythrocyte suspensions were incubated with 1,6-diphenyl-1,3,5-hexatriene (DPH) or 1-(4-trimethylammoniumphenyl)-6-phenyl-1,3,5-hexatriene p-toluenesulfonate (TMA-DPH) (Invitrogen, Eugene, OR, USA) (final concentration = 1 µM) for 10 min [32], and exposed to the SMFs for 30 min at room temperature. Fluorescence intensities of DPH and TMA-DPH were then measured at an excitation wavelength (*λ_ex_*) of 360 nm and an emission wavelength (*λ_em_*) of 430 nm using a polarization fluorometer (Chameleon; Hidex Inc, Turku, Finland). Emission intensity was determined through a polarizing filter both plane parallel (*I_vv_*) and perpendicular (*I_vh_*) to the excitation polarizing filter. All measured intensities were corrected for light-scattering effects using unlabeled erythrocyte suspensions. Finally, the fluorescence anisotropy (*r*) was calculated using the following formula [33]: 
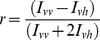



### Dehydration stability test

For estimating the dehydration injury of freezing on the erythrocytes, hypertonic saline was reported to be a useful method by several researchers [13], [34]. In brief, the erythrocyte samples were washed and suspended in normal saline, then the erythrocyte suspensions were exposed to SMFs at room temperature for 30 min. To simulate the dehydration phenomenon during freezing, SMF-exposed erythrocyte suspensions were added to a hypertonic saline solution resulting in a final saline concentration of 18% (w/v). After 5 min incubating at room temperature, the erythrocyte-saline suspensions were centrifuged at 1,200× *g* for 5 min and used in the hemolysis test. For normalizing results, the relative hemolysis was defined as the ratio between the hemolysis percentages of the SMF-exposed sample and the control sample from the same volunteer.

### Statistics

All data are expressed as medians ± interquartile ranges (IQR). For statistical tests, a Friedman test with Bonferroni correction was used to evaluate the significance of differences among groups, and *p* values less than 0.05 were considered statistically significant.

## Results

### Survival after freezing and thawing

**Figure 2:**
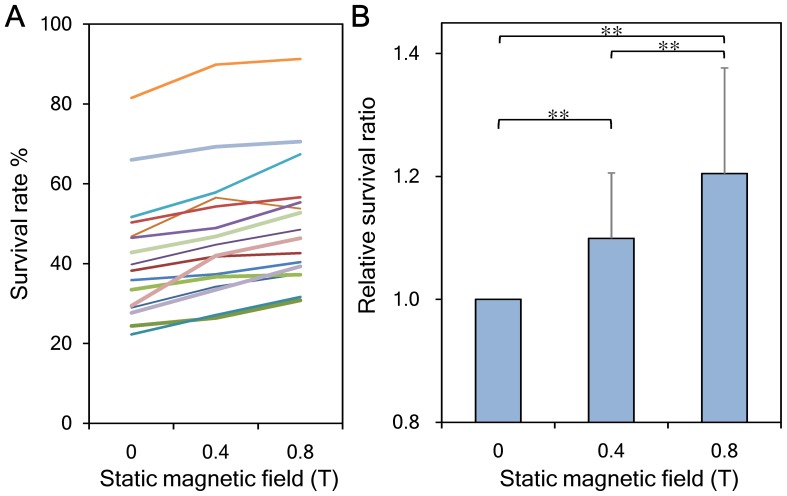
Survival rates of the thawed erythrocytes slowly frozen in different magnetic flux densities. (**A**) For all the 16 samples, the survival rate (%) increased as the SMF flux density increased. (**B**) From the normalized data, erythrocytes exposed to 0.4-T and 0.8-T SMFs during the freezing process had significantly increased relative survival ratios by 10% and 20% respectively. (^**^ *p*<0.001)

Before the freezing process, the mean Hct of the glycerolized erythrocyte samples was 32%. Figure 2A shows the survival rates of thawed erythrocytes that were frozen with SMFs and stored at −80°C for 24 hr. For each volunteer's sample, the increased magnetic flux density of the SMF exposure resulted in a significant positive effect on thawed erythrocyte survival rate (*p*<0.001). When the figure was replotted using the normalized data (Figure 2B), significant increases of 10% and 20% were found when the 0.4-T and 0.8-T SMFs were applied in the freezing procedure respectively.

### Morphology studies

**Figure 3:**
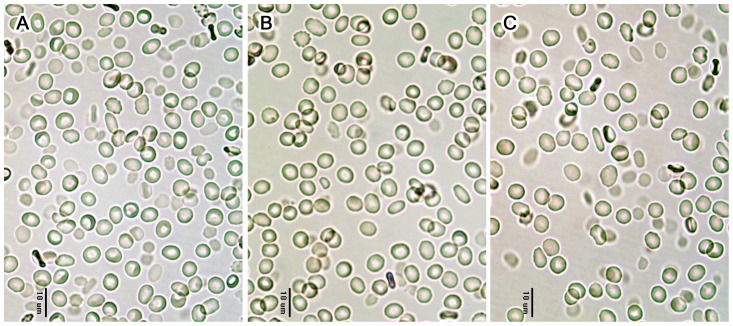
Micrographs of the thawed erythrocytes slowly frozen in static magnetic fields. No significant difference are visible for erythrocyte samples thawed after freezing coupled with (**A**) 0-T SMF, (**B**) 0.4-T SMF, and (**C**) 0.8-T SMF. Original magnification ×400, bars represent 10 µm.

**Figure 4:**
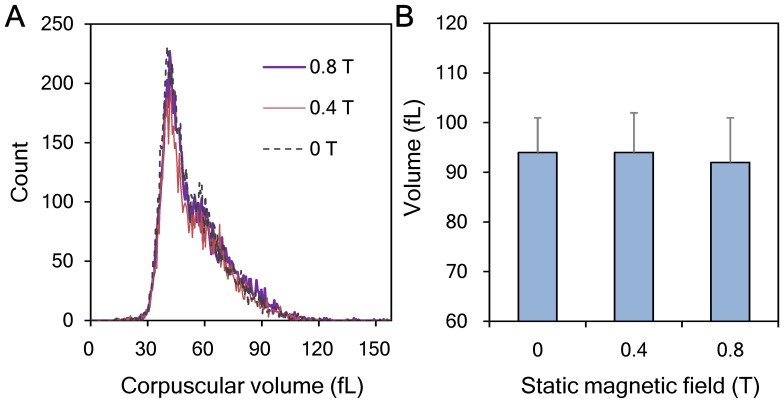
Corpuscular volume analysis of the thawed erythrocyte subjected to SMF-coupled slow freezing. No significant difference was found among the three groups erythrocytes subjected to the various flux densities of static magnetic fields during freezing. (**A**) Corpuscular volume distribution. (**B**) Mean corpuscular volumes (MCV).

To understand the morphologic changes of erythrocytes subjected to SMF exposure during freezing, thawed erythrocytes of control and experimental groups were observed using an optical microscope. As shown in Figure 3, thawed erythrocytes in the 0.4-T (Figure 3B) and 0.8-T (Figure 3C) groups maintained their typical biconcave disk shape and showed no visible change when compared to the control group (Figure 3A). In addition, no obvious anisocytes or aggregated erythrocytes were found in the thawed samples. Corpuscular volume distributions of the thawed erythrocytes overlapped closely, as can be seen in Figure 4A. Additionally, the mean corpuscular volume (MCV) of each group was about 93 fL, with no significant difference found between groups (Figure 4B).

### Erythrocyte metabolites

**Figure 5:**
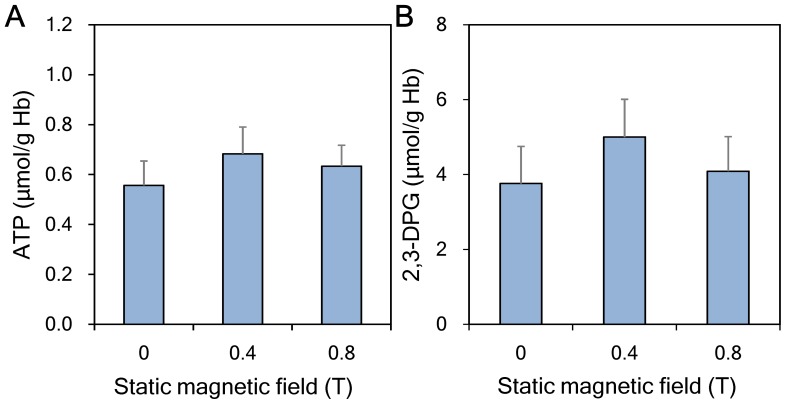
Metabolite concentrations of erythrocytes thawed after slow freezing coupled with various magnetic flux densities. No significant difference was found among the three static magnetic field groups for (A) ATP and (B) 2,3-DPG.

To test whether the SMF-freezing process had negative effects on the function of erythrocytes, ATP and 2,3-DPG concentrations of the thawed erythrocytes were determined. We found the SMF-freezing process had no effect on ATP (Figure 5A) or 2,3-DPG concentrations (Figure 5B).

### Membrane fluidity test

**Figure 6:**
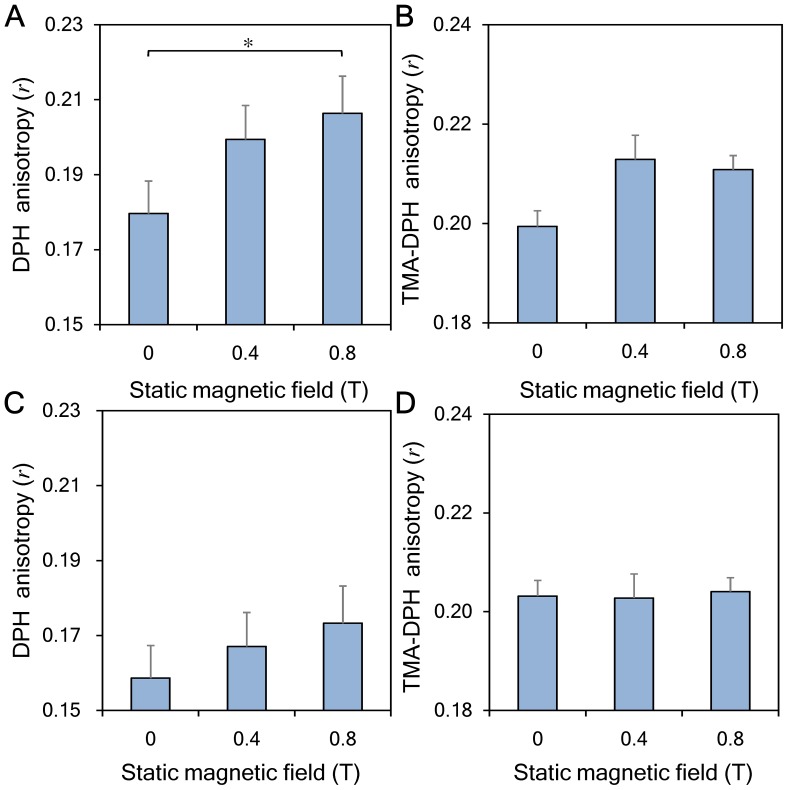
Measurements of fluorescence anisotropies (*r*) of erythrocytes exposed to 0, 0.4, and 0.8-T SMFs. (**A**) Erythrocytes exposed to a 0.8-T SMF for 30 min showed significantly increased DPH-fluorescence anisotropy values. (**B**) A slight increase in TMA-DPH-fluorescence anisotropy was found when erythrocyte samples were exposed to a 0.8-T SMF for 30 min. When the blood cells were removed from the SMF for 15 min, the measured (**C**) DPH and (**D**) TMA-DPH fluorescence anisotropy recovered to their basal levels for both SMF-exposed groups. (^*^ *p*<0.05)

Cell membrane fluidity of the erythrocyte samples exposed to SMFs was determined by assaying fluorescence anisotropy at room temperature. The fluorescence probes DPH and TMA-DPH were respectively incorporated in the hydrophobic and hydrophilic regions of phospholipid bilayer. The results showed that DPH fluorescence anisotropies (*r*) increased when the magnetic flux density was increased. The *r* values differed significantly between the 0.8-T group (*r* = 0.2063±0.0320) and the control group (*r* = 0.1796±0.0241) (Figure 6A). Similar results were found in TMA-DPH experiment. As shown in Figure 6B, the *r* value increased slightly from 0.1994±0.0054 (0 T, control) to 0.2108±0.0048 (0.8-T group). However, after erythrocyte samples were removed from the SMFs for more than 15 min, there were no statistically significant differences found in either the DPH or TMA-DPH experiments (Figure 6C and 6D).

### Dehydration stability test

**Figure 7:**
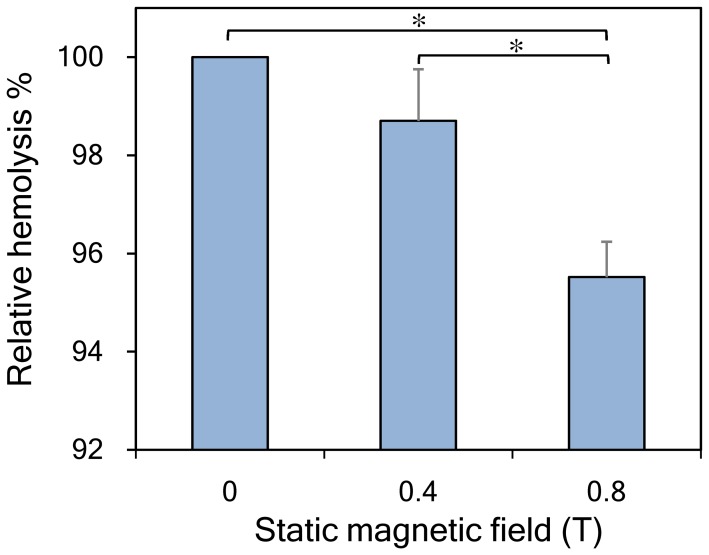
Dehydration stability tests of erythrocyte suspensions exposed to static magnetic fields for 30 min. Erythrocyte samples exposed to a 0.8-T SMF had significantly decreased relative hemolysis. Low hemolysis value in this figure represents a better dehydration stability of the erythrocyte sample. (^*^ *p*<0.05)

To test the effect of the SMFs on the membrane stability during dehydration, the erythrocyte samples were incubated in highly concentrated saline at room temperature. The 0.8-T SMF-exposed samples exhibited significantly lower (*p*<0.05) hemolysis when compared to the control group (Figure 7). In other words, cell membranes in the 0.8-T SMF-exposed samples demonstrated greater resistance to extreme dehydration.

## Discussion

The aim of this study was to investigate the cryopreservative efficiency of erythrocytes frozen in the presence of an SMF using a slow cooling procedure. Our results showed that the use of a 0.8-T SMF during the freezing process increased the relative survival ratio of frozen-thawed erythrocytes by 20% compared to the 0-T, sham-exposed group (Figure 2B). To ensure that SMF exposure can be used as an adjuvant technique for erythrocyte cryopreservation, possible harm due to SMFs should be evaluated.

Cell morphology is an important parameter for evaluating the health status of erythrocytes. A previous study reported that erythrocytes exposed to the strong SMF of an MRI scanner for a period of 18 min can change erythrocyte morphology and aggregation status. However all the changes were reversible when erythrocytes were removed from the SMF source [29]. We also found that the SMF effects on the physical properties of erythrocyte cell membranes were reversible after removing erythrocytes from the SMF source for 15 min (Figure 6). These results may explain the finding that erythrocytes thawed from an SMF-coupled freezing process demonstrated no obvious change in morphology (Figure 3) or corpuscular volume (Figure 4). Cell aggregation is another important indicator for assessing the status of SMF-exposed erythrocytes. Erythrocytes thawed from the SMF-coupled freezing process used in this study exhibited no obvious cell aggregation (Figure 3). Previous studies showed that when erythrocytes were immersed in a normal saline solution with an Hct of 20%, the effect of an SMF on erythrocyte aggregation was slight, even when the cells were exposed to a strong SMF of up to 6.3 T [35], [36]. Additionally, ATP and 2,3-DPG metabolite tests were used to assess erythrocyte function after thawing [31]. We found that neither ATP nor 2,3-DPG measurements exhibited any obvious delayed magnetic effect (Figure 5). Since we found no negative effect on the ATP content of the tested erythrocytes, SMFs would not enhance energy-dependent apoptosis or necrosis of erythrocytes, which are considered the secondary causes of post-thaw erythrocyte death [11].

The study of electron spin resonance demonstrates that cell membranes with low fluidity exhibit lower permeability [37]. In addition, fluorescence anisotropy is inversely related to membrane permeability [38]. Previous investigations show that cells exposed to SMFs have decreased cell membrane fluidity [25], [27]. Thus, it is reasonable to infer that erythrocyte membrane permeability and membrane elasticity can be significantly decreased by the SMF of an MRI scanner [29]. In this study, membrane fluidity was determined by fluorescence anisotropy (*r*). As shown in Figure 6A and 6B, exposing erythrocytes to a 0.8-T SMF for 30 min decreased membrane fluidity. In addition, the fluorescence anisotropies increased in both the hydrophobic and hydrophilic regions of the cell membranes, with a ratio of 115% and 106%. Therefore, the SMFs used in this study could decrease the erythrocyte membrane fluidity, and this phenomenon could result in reduced erythrocyte membrane permeability.

It is well known that dehydration is the major cause of cryo-injury when cells are subjected to slow cooling procedures. The dehydration effect induces stress on the cell membrane during freezing. These stresses produce physical deformation and phase behavior change, which results in damage to the cell membrane [10]. In 2005, Blesbois *et al* [39] performed an experiment to test cryopreserved bird semen. They found that spermatozoa with low membrane fluidity had better survival rates after cryopreservation. This result suggests that low-fluidity membranes of the spermatozoa gave higher resistance to freezing-induced stresses [39]. Our results also confirm this finding (Figure 7). In the dehydration stability test, SMF-exposed erythrocytes exhibited greater membrane stability and resistance to the extremely dehydrating environment (Figure 7). Thus, it is reasonable to suggest that the 0.8-T SMF exposure decreased the membrane fluidity of the erythrocytes and resulted in two possible mechanisms helpful for erythrocyte cryopreservation. First, the decrease in membrane fluidity results in a reduction in basal membrane permeability, thus decreasing water transportation across the cell membrane during the slow cooling procedure. Second, the increase in membrane rigidity has a positive effect on membrane resistance to stresses caused by excessive dehydration.

It has been reported by several investigators that membrane permeability is affected by freezing-induced membrane phase transition [10], [40]. Although the addition of cryoprotectant does not prevent liquid-crystal-to-gel membrane phase changes during freezing, the presence of cryoprotectants can decrease the nucleation temperature. The addition of cryoprotectant causes membrane phase transition to occur more gradually and over a wider temperature range [41], [42]. This phenomenon means that the cellular membrane has more time to transport water causing cell dehydration. In our system, we also found that the addition of a 20% glycerol solution reduced the nucleation temperature to −18 to −20°C (Figure 1D). The use of this cryoprotectant allows for both gradual membrane phase transition and the prolongation of the liquid crystal phase to occur at sub-zero temperatures. Since the effect of SMF on cellular membranes is the reorientation of liquid-crystal molecules, such as phospholipids [22]–[24], using a cryoprotectant to allow gradual membrane phase transition and to prolong the liquid-crystal phase should increase the affective time of SMF at subzero temperatures.

There is no evidence that either water molecules or hemoglobin exposed to SMFs have any effect on erythrocytes in the low-temperature environment of the freezing process. Water molecules are small particles expressing diamagnetic susceptibility. It was reported that a magnetic flux density approaching 450 T causes water molecules to change orientation by the negligible amount of 0.01% [43]. In addition, although hemoglobin is a molecule enriched with iron that exhibits a small paramagnetic moment [24], the effect of magnetic fields on hemoglobin is less than thermal energy [26].

In conclusion, we found that the SMF coupled with the slow cooling procedure increased survival rates of frozen-thawed erythrocytes without any negative effects on cell morphology or function. We suggest that the SMF cryoprotective effect is due to enhanced biophysical stability of the cell membrane, which reduces dehydration damage to the erythrocyte membrane during the slow cooling procedure.
